# The Role of Thrombelastography in Multiple Trauma

**DOI:** 10.1155/2011/895674

**Published:** 2011-09-07

**Authors:** Victor Jeger, Heinz Zimmermann, Aristomenis K. Exadaktylos

**Affiliations:** Department of Emergency Medicine, University Hospital Inselspital, 3010 Bern, Switzerland

## Abstract

Hemorrhage and traumatic coagulopathyis are major causes of early death in multiply injured patients. Thrombelastography (TEG) seems to be a fast and accurate coagulation test in trauma care. We suggest that multiply injured trauma patients would benefit the most from an early assessment of coagulation by TEG, mainly RapidTEG, to detect an acute traumatic coagulopathy and especially primary fibrinolysis, which is related with high mortality. This review gives an overview on TEG and its clinical applications.

## 1. Introduction

Hemorrhage is a major cause of early death in multiply injured patients. One of the reasons of uncontrolled hemorrhage may be acute traumatic coagulopathy. It has been first discussed by Brohi and colleagues, and it is now thought to be induced by trauma and hypoperfusion [1, 2]. The pathomechanism of acute traumatic coagulopathy is extensively reviewed by Hess et al. [3] 25% of major trauma patients suffer from coagulopathy at admission to the hospital, and its presence is associated with a fourfold increase in mortality [2].

The initial treatment of bleeding trauma patients is not limited any more to damage control surgery but to damage control resuscitation, using a balanced administration of blood products in the ratio of red blood cells: fresh frozen plasma as 1 : 1 or 1 : 2, which is able to correct hypovolemia, anemia, and, to a certain degree, the acute traumatic coagulopathy [4, 5]. Additionally, the acute traumatic coagulopathy may also occur in absence of acute bleeding, for example, due to massive blunt injury and hypothermia. This pattern is typical for our patients population in a Level 1 trauma center in Switzerland, where we face mainly car accidents and injuries related to outdoor sports (skiing, climbing, base jumping, avalanches, etc.). In situations where coagulopathy is frequent but less obvious at admission of the patient to the resuscitation bay, the decisions should rely on evidence based point of care devices to correct coagulopathy. In reality, the trauma physician is somehow blinded to the current state of coagulation because of long turnover times of standard coagulation screening from the lab and he/she has to base decisions on experience and gut feeling [6].

The search for appropriate point of care devices in trauma care brought thrombelastography (TEG) back in focus in 1997 by Kaufmann et al. after the technique had been used for years in cardiac and liver surgery [7, 8]. 

## 2. Thrombelastography—Assessing the Viscoelastic Properties of the Thrombus

**Figure 1:**
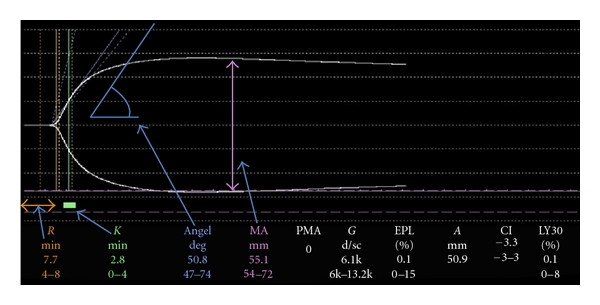
Normal TEG tracing with arrows highlighting the most important TEG parameters (*r*, *K*, angle and MA).

The concept of thrombelastography had been first described by Hartert in 1948 [9]. Only its development to a compact device in combination with a computer made its manipulation easier and its measurements reproducible. The technique had been described before and should only be mentioned briefly [7, 10, 11]. A small volume of blood (340–360 *μ*L) is placed in an oscillating cup kept at 37°C or at the patient's current temperature. A pin is suspended in the cup from a torsion wire with an electrical transducer. Initially there is no clot, the motion of the cup does not affect the pin, and a straight line trace is obtained. As the blood in the cup clots however, the motion of the rotating cup is transmitted to the pin and its oscillation is recorded (Figure 1) [11]. The procedure can be accelerated by activating coagulation of the sample with kaolin, a celite, which increases the surface (intrinsic activation), or with tissue factor (extrinsic activation, RapidTEG). Additionally, heparinase coated cups and pins can be used to monitor coagulation properties of the patient's blood underneath heparin therapy. Finally, there are also kits to measure platelet function or fibrinogen in special situations, which are not discussed in this paper.

The most important TEG parameters are the reaction time “*r*”, which measures the initiation of coagulation until a first pin oscillation of 2 mm and represents initiating coagulation factors, “*K*” and “alpha angle” represent the dynamic of the clot formation and are measures of time from the end of “*r*” to 20 mm of pin oscillation, and the slope between “*r*” and “*K*” respectively. They correspond mainly with fibrinogen concentration. The maximal amplitude (MA) represents clot strength and is therefore an estimation of platelet function. Finally, LY30, which is the lysis of the clot 30 minutes after MA, measures the rate of fibrinolysis. These parameters reflect the strength of thrombelastography to monitor not only one step of the cascade-like conventional coagulation tests, which focus only on fibrin formation. It gives a rapid overview on the main players in the cascade which are important to the trauma physician like initiation factors, fibrinogen, platelet function, and fibrinolytic components.

As mentioned above, TEG has evolved in an automated device which only needs 2-3 pipetting manipulations and not more than 2 minutes to start. It is possible to train physicians and nurses in only one or two short sessions, even with no previous lab experience, to run a TEG accurately. A step by step protocol, as provided by the manufacturer, is helpful, especially in smaller trauma units, where TEG is not performed every day. Quality control test runs should be performed regularly and may be attributed to the quality control experts for point-of-care devices of your hospital. The site of blood sampling seems not to be neglectable, especially when comparing patients in a study or if there is repeated measurements on the same patient. It has been shown that there are differences between the sampling sites, not related to the oxygen content but to the calculated shear forces in the corresponding blood vessel [12].

Two different but very similar devices exist to measure thrombelastography: TEG 5000 Analyzer from Haemonetics (Braintree, MA, USA) and ROTEM from TEM (Munich, Germany). This paper did focus specially on the TEG device, but the conclusions from the current literature are applicable for ROTEM as well.

## 3. Animal Studies—Trauma Models and Coagulopathy

In trauma research, the main limitation, even in large multicenter studies, is the high variability of the studied patient sample. There is a heterogeneity in the injury pattern, age, comorbidities, mechanism of injury, and in many more. This is the reason why trauma studies focusing on hemodynamics, hemorrhage control, and coagulopathy are performed with animal models. Some recent publications are mentioned here, which all showed that TEG is a reasonable coagulation test in the early assessment of trauma patients. 

That pigs are a good model for research in coagulation and fibrinolysis has been shown by Velik-Salchner and colleagues [13]. White et al. worked with a pig model for traumatic hemorrhagic shock consisting of soft tissue injury and femur fracture. They randomized 23 pigs in 18 hemorrhage and 5 controls. With the beginning of hemorrhagic shock, fibrinogen concentration decreased rapidly. With increasing shock, TEG MA was reduced from 68.8 (SE: 0.9) to 64.7 (SE: 0.9). All other TEG parameters and conventional coagulation tests remained unchanged during hemorrhage [14]. Martini and colleagues were interested in the effect of hypothermia or hemorrhage on coagulation. Therefore, they randomized 24 pigs in four groups (control, hemorrhage with resuscitation, hypothermia, hemorrhage with resuscitation, and hypothermia). PT and aPTT did not change by hemorrhage or hypothermia. However, TEG parameters were affected by both: hypothermia prolonged *r*-time and *K* and decreased the angle alpha, hemorrhage only decreased MA. As only TEG was able to differentiate the mechanism of coagulopathy, the authors suggested, that TEG may be a suitable test to guide treatment of clotting alterations associated with hypothermia and hemorrhagic shock [15]. Kheirabadi et al. had a similar conclusion in a rabbit model of coagulopathy: 21 rabbits were randomized in three groups (control, warfarin treated, hemodiluted hypothermia). All animals underwent splenic injury. PT was valid to monitor warfarin-induced coagulopathy but failed to be reliable as a screening test for dilutional and hypothermic coagulopathy. TEG measurements of blood clotting rate represented better coagulopathic bleeding and mortality [16]. Finally, in a very complex pig model of multiple injury, preclinical phase and clinical phase, Alam and colleagues induced an acute traumatic coagulopathy. Different protocols of resuscitation had an effect on coagulation, which was measured most accurately with TEG: Hetastarch worsened coagulopathy, but it was rapidly reversed with the administration of blood components, especially FFP [17].

## 4. Clinical Studies—Describing the Trauma Patient

Kaufmann and colleagues studied the use of TEG in 69 blunt trauma patients and found that only Injury Severity Score (ISS) and TEG were predictive for transfusion. They described that the majority of their patients studied was hypercoagulable [8]. Schreiber et al. did also discover that their trauma patients (*n* = 65) were hypercoagulable. They used noncitrated whole blood for the kaolin-activated TEG measurements. Mainly on day 1 (62%) compared to day 4 (26% of patients) after admission to the hospital. Women were more hypercoagulable than men. Like in animal studies described above, the authors did not detect pathologic conventional coagulation test values, which were within normal limits throughout the study [18]. However, the finding that trauma patients show mainly hypercoagulable changes had not been found by others. One explanation may be the small sample size and the high variability among trauma patients. Rugeri et al. used the ROTEM device in their trauma study (*n* = 88), which uses citrated whole blood. Four consecutive measurements had been done over the first 24 hours after admission to the ER. One third of the patients presented a coagulopathy at the first measurement after admission to ER. The authors showed strong correlations of conventional coagulation screening with the corresponding ROTEM parameter. However, the authors did not show the data of the evolution of ROTEM values over time, as they pooled all the ROTEM values together to compare and correlate them with the corresponding conventional coagulation parameter [19].

A larger sample of trauma patients (*n* = 161) had been studied by Carrol and colleagues: TEG had been performed either tissue factor activated or using platelet mapping. One sample was collected onsite and the second in the emergency department (ED). The authors did not detect changes from onsite to ED. No differences had been found between 22 patients who received blood products compared to the other patients. Only by using the platelet mapping assay, differences in adp responsiveness were obtained. When they had a closer look to the 14 fatalities in their sample, they found a difference in r value and MA compared to survivors. Hyperfibrinolysis was detected in three patients of whom two died. The authors concluded that TEG should be used as an additional tool to standard coagulation tests, especially to monitor hyperfibrinolysis, which is related to high mortality [20].

TEG had also been evaluated and applied in combat support hospitals by the U.S. Army in Iraq and Afghanistan. Plotkin et al. described 44 patients with combat injuries. A TEG was run with noncitrated whole blood samples (kaolin activated) within 24 hours after admission to the hospital. Conventional coagulation tests were suggesting hypocoagulation but did not correlate with blood product use. However, TEG values, especially MA correlated with blood product use platelet count [21].

To evaluate the best ratio of packed blood cells (PBC) to fresh frozen plasma (FFP), Davenport et al. used a ROTEM device to assess the coagulation status of 50 trauma patients who received more than 4 units PBC. The authors stated that a ratio of 1 : 2 or 3 : 4 should be achieved to preserve coagulation and a ratio of 1 : 1 did not show to be better. However, if the ratio is <1 : 2, patients presented hypocoagulable ROTEM results [5].

We started a few years ago to introduce tissue factor activated TEG (RapidTEG) in our resuscitation bay of a level 1 trauma center in Switzerland. We found that using noncitrated whole blood from samples drawn at admission of the patient to resuscitation bay provided the trauma physician with fast and accurate results about the patient's coagulation status. This may help to guide focused blood product administration early in the treatment [11]. Kashuk and colleagues were studying the use of RapidTEG in trauma as well. In a first study (*n* = 44), they were interested if the use of noncitrated whole blood would have advantages compared to citrated whole blood. The authors correlated RapidTEG results with corresponding conventional coagulation measurements and found slightly higher correlation between noncitrated TEG values with CCT compared to citrated TEG results. This led the authors to the assumption that noncitrated samples may be more accurate. However, they stated that it is difficult to compare a static (conventional) with a dynamic test (TEG) [22]. In a further study, analyzing the data of 61 trauma patients, Kashuk et al. found that 34% of patients requiring massive transfusion suffered from primary fibrinolysis. 64% of these patients sustained penetrating injuries.RapidTEG tests had been run with noncitrated whole blood. The authors suggested the G-value as a measure of clot strength, (dynes/cm^2^) as a predictive parameter for identification of fibrinolysis, and death as early as one hour after injury.

## 5. Conclusion

According to the current literature, thrombelastography seems to be a fast and accurate coagulation test in trauma care. We suggest that multiply injured trauma patients with an ISS > 16 would benefit the most from an early assessment of coagulation by TEG, mainly RapidTEG, to detect an acute traumatic coagulopathy and especially primary fibrinolysis, which is related with high mortality. Where the TEG should be run depends on the staff resources of your emergency department. We suggest to draw citrated whole blood at the admission of the trauma patient to the resuscitation bay and to send it to the central hematologic lab to run the test. The results may then be presented on a screen at the bed side. Tieu et al. did also share this opinion and stated that it may be difficult to run a TEG on noncitrated whole blood, especially due to the handling time of less than 4 minutes after blood sampling. This may demand multiple TEG devices in many critical areas of a hospital. Although some effects of citrated blood on TEG values had been observed, it may be an alternative to run a TEG on citrated whole blood in a central lab like conventional coagulation tests [23]. This may prevent the trauma physicians from an additional manipulation and could give them TEG values with a better quality control and reproducibility.

Interventional multicenter studies are needed to establish the use of TEG in the trauma setting. May early TEG results influence blood product administration and would this change the outcome of trauma patients? Do we use the correct TEG transfusion thresholds? Hopefully, we are able to answer some of these questions soon to improve the outcome of trauma patients.
